# High leukocyte mtDNA content contributes to poor prognosis through ROS-mediated immunosuppression in hepatocellular carcinoma patients

**DOI:** 10.18632/oncotarget.8071

**Published:** 2016-03-14

**Authors:** Xianli He, Falin Qu, Feng Zhou, Xingchun Zhou, Yibing Chen, Xu Guo, Jibin Li, Qichao Huang, Yefa Yang, Zhuomin Lyu, Hongxin Zhang, Jinliang Xing

**Affiliations:** ^1^ Department of General Surgery, Tangdu Hospital, The Fourth Military Medical University, Xi'an 710032, China; ^2^ State Key Laboratory of Cancer Biology and Experimental Teaching Center of Basic Medicine, The Fourth Military Medical University, Xi'an 710032, China; ^3^ Department of Radioactive Intervention, Eastern Hepatobiliary Surgery Hospital, The Second Military Medical University, Shanghai 200438, China; ^4^ Department of Pain Treatment, Tangdu Hospital, The Fourth Military Medical University, Xi'an 710032, China

**Keywords:** mitochondrial DNA content, hepatocellular carcinoma, prognosis, reactive oxygen species, immunosuppression

## Abstract

Compelling epidemiological evidences indicate a significant association between leukocyte mitochondrial DNA (mtDNA) content and incidence risk of several malignancies, including hepatocellular carcinoma (HCC). However, whether leukocyte mtDNA content affect prognosis of HCC patients and underlying mechanism has never been explored. In our study, leukocyte mtDNA content was measured in 618 HCC patients and its prognostic value was analyzed. Moreover, we detected the immunophenotypes of peripheral blood mononuclear cells (PBMCs) and plasma concentrations of several cytokines in 40 HCC patients and assessed the modulating effects of mtDNA content on immunosuppression in cell models. Our results showed that HCC patients with high leukocyte mtDNA content exhibited a significantly worse recurrence-free survival (RFS) and overall survival (OS) than those with low leukocyte mtDNA content. Leukocyte mtDNA content and TNM stage exhibited a notable joint effect in prognosis prediction. Furthermore, we found that patients with high leukocyte mtDNA content exhibited a higher frequency of CD4^+^CD25^+^FOXP3^+^ regulatory T (Treg) cells and lower frequency of NK cells in PBMCs and had higher TGF-β1 and lower TNF-α and IFN-γ plasma concentration when compared with those with low leukocyte mtDNA content, which suggests an immunosuppressive status. High leukocyte mtDNA content significantly enhanced the ROS-mediated secretion of TGF-β1, which accounted for higher Treg and lower NK frequency in PBMCs. In a conclusion, our study for the first time demonstrates that leukocyte mtDNA content is an independent prognostic marker complementing TNM stage and associated with an ROS-mediated immunosuppressive phenotype in HCC patients.

## INTRODUCTION

Hepatocellular carcinoma (HCC) is the sixth most common malignant disease and the third leading cause of cancer-related death worldwide [1]. Despite recent improvements in diagnosis and treatment, clinical outcome of HCC is still disappointing [2]. Due to the molecular and genetic heterogeneity, HCC patients with the similar clinical and pathological features often exhibit distinct outcomes [3]. Therefore, it is urgent to identify new molecular biomarkers to complement TNM staging system for more precise prognostic prediction of HCC, and thus benefit for individualized therapy.

Mitochondria plays multiple roles in energy metabolism and cellular homeostasis, mainly including the generation of ATP and reactive oxygen species (ROS) [4]. Human mitochondrial DNA (mtDNA) is a circular double-stranded DNA molecule of 16,569 base pairs [5]. The mtDNA content (also referred as mtDNA copy number) varies from several hundred to more than 10,000 copies per cell, depending on the cell type [6]. The mtDNA content may undergo significant changes under diverse internal or external microenvironments, which can lead to impairment of the OXPHOS system and the enhanced generation of ROS. This scenario has been proposed to contribute to the initiation and progression of tumors [7].

Quantitative changes in mtDNA content have been observed in many types of malignancies, such as HCC, gastric cancer, head and neck cancer and colorectal cancer (CRC) [8–11]. Previous studies have also revealed that altered mtDNA content in tumor tissues was associated with tumor stage, prognosis, and treatment response, again in a cancer type-specific manner [10, 12–14]. Yamada et al. have found that HCC tissues have a reduced copy number of mtDNA when compared with paired non-tumor tissues, which is associated with malignant potential of HCC [15]. In recent years, there are a series of reports on the association of the mtDNA content in peripheral blood lymphocytes (PBLs) with cancer susceptibility [16–20]. However, only two studies have indicated that mtDNA content in PBLs is associated with breast cancer progression and CRC prognosis [21, 22]. The effect of mtDNA content in PBLs on HCC patient prognosis and its underlying mechanism has not been explored.

Herein, we measured mtDNA content in PBLs from HCC patients and assessed its prognostic value. Furthermore, we explored the potential immune-related mechanism underlying the prognostic effects of leukocyte mtDNA content. To the best of our knowledge, this is the first study to investigate the prognostic significance of leukocyte mtDNA content in HCC patients, which may benefit for future improvement of treatment.

## RESULTS

### Characteristics of patient populations and distribution of mtDNA content

Demographic and clinical characteristics of HCC patients were summarized in Supplementary Table 1. At latest follow-up, 373 patients developed recurrence and 240 died. The median value (range) of normalized mtDNA content was [0.98 (0.12-3.70)] in total samples and no significant difference was observed for mtDNA content among training [0.99 (0.16-3.39)] and validation [0.98 (0.12-3.70)] cohorts (*P* = 0.987) (Supplementary Table 1). We then compared mtDNA content between various epidemiological and clinical characteristics in training cohort, validation cohort and total patients (Supplementary Table 2). No significant difference of mtDNA content was found between patients with different age, sex, HBsAg status, tumor size, number of tumors, PVT, TNM stage, differentiation, and AFP level (*P* values ranging from 0.092 to 0.996). However, mtDNA content was significantly higher in patients with recurrence than those in patients without recurrence in total cohort (*P* < 0.001). Similar result was observed in patients who died when compared with those who were alive (*P* = 0.001).

### Prognostic analysis for leukocyte mtDNA content in HCC patients

**Figure 1:**
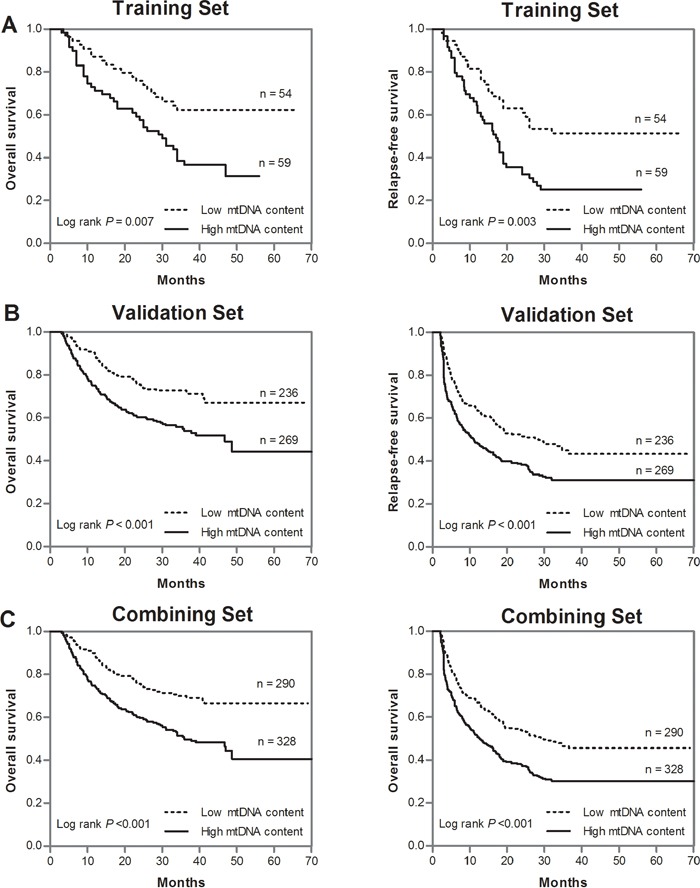
Kaplan-Meier estimates of OS and RFS in HCC patients by leukocyte mtDNA content **A.** OS and RFS in the training cohort; **B.** OS and RFS in the validation cohort; **C.** OS and RFS in the total cohort.

To find the optimal cutoff point of mtDNA content value that best distinguish the OS and RFS for HCC patients, we constructed the ROC curves for events based on mtDNA content values in the training cohort and found a mtDNA content value of 0.98 to be the best cutoff points for OS and RFS (*P* = 0.004 and 0.002, Supplementary Figure 1). This value was used as a uniform cutoff point to dichotomize patients into two subgroups with high or low mtDNA content in all the subsequent analyses. Then, we analyzed the prognostic effect of mtDNA content on OS and RFS. Univariate (Supplementary Table 3) and multivariate analyses (Table 1) indicated that high leukocyte mtDNA content was significantly associated with poor OS and RFS of patients in training cohort (HR = 1.89, 95%CI = 1.05 - 3.39, *P* = 0.034; HR = 1.90, 95%CI = 1.12 - 3.22, *P* = 0.018, respectively) and validation cohort (HR = 1.88, 95%CI = 1.38 - 2.58, *P* < 0.001; HR = 1.84, 95%CI = 1.22 - 2.95, *P* < 0.001, respectively). Combining two patient cohorts, high leukocyte mtDNA content was associated with a 1.89-fold increased risk of death (95% CI, 1.44 - 2.48) and 1.86-fold increased risk of recurrence (95% CI, 1.26 - 2.98), respectively (Table 1). Kaplan-Meier survival function analysis showed that patients with high mtDNA content had a shorter OS and RFS than did those with low mtDNA content in the training (log-rank *P* = 0.007 and 0.003, respectively, Figure 1A), validation (both log-rank *P* < 0.001, Figure 1B) and total cohort (both log-rank *P* < 0.001, Figure 1C).

**Table 1:** Multivariable Cox regression analysis of prognosis for HCC patients

Variables	Training cohort		Validation cohort		Total cohort	
	HR (95% CI)	*P*value	HR (95% CI)	*P*value	HR (95% CI)	*P*value
**Overall survival**						
Age (>52 *vs.* ≤52)	1.34 (0.77 - 2.33)	0.305	1.13 (0.83 - 1.54)	0.443	1.13 (0.86 - 1.48)	0.368
Sex (male *vs.* female)	1.19 (0.36 - 3.99)	0.774	1.14 (0.71 - 1.85)	0.582	1.29 (0.83 - 1.99)	0.253
HBsAg (postive *vs.* negative)	1.69 (0.83 - 3.47)	0.149	1.14 (0.65 - 1.99)	0.657	1.19 (0.84 - 1.68)	0.339
TNM stage (III+IV *vs.* I+II)	3.01 (1.63 - 5.58)	**<0.001**	2.29 (1.55 - 3.37)	**<0.001**	1.74 (1.21 - 2.51)	**0.003**
Differentiation (Poor *vs.* Moderate+Well)	2.55 (1.47 - 4.93)	**<0.001**	2.11 (1.25 - 3.69)	**<0.001**	1.98 (1.17 - 3.08)	**0.008**
Serum AFP (≥200μg/L *vs.* <200μg/L)	2.42 (1.31 - 4.45)	**0.005**	1.81 (1.31 - 2.49)	**<0.001**	1.73 (1.31 - 2.28)	**<0.001**
mtDNA content (high *vs.* low)	1.89 (1.05 - 3.39)	**0.034**	1.88 (1.38 - 2.58)	**<0.001**	1.89 (1.44 - 2.48)	**<0.001**
**Recurrence-free survial**						
Age (>52 *vs.* ≤52)	1.08 (0.66 - 1.78)	0.75	0.98 (0.77 - 1.25)	0.874	1.00 (0.81 - 1.24)	0.99
Sex (male *vs.* female)	1.12 (0.39 - 3.16)	0.836	1.26 (0.87 - 1.83)	0.225	1.31 (0.93 - 1.85)	0.125
HBsAg (postive *vs.* negative)	1.55 (0.82 - 2.96)	0.181	1.02 (0.67 - 1.54)	0.93	1.17 (0.75 - 1.81)	0.496
TNM stage (III+IV *vs.* I+II)	4.12 (2.36 - 7.20)	**<0.001**	1.83 (1.34 - 2.51)	**<0.001**	1.72 (1.28 - 2.32)	**<0.001**
Differentiation (Poor *vs.* Moderate+Well)	2.29 (1.38 - 3.85)	**<0.001**	2.06 (1.25 - 3.84)	**<0.001**	1.97 (1.17 - 2.93)	**0.009**
Serum AFP (≥200μg/L *vs.* <200μg/L)	1.84 (1.11 - 3.04)	**0.018**	1.61 (1.25 - 2.07)	**<0.001**	1.61 (1.29 - 2.02)	**<0.001**
mtDNA content (high *vs.* low)	1.90 (1.12 - 3.22)	**0.018**	1.84 (1.22 - 2.95)	**<0.001**	1.86 (1.29 - 2.98)	**<0.001**

Notes: mtDNA, mitochondrial DNA; HR: hazard ratio; 95% CI, 95% confidence interval. Significant *P* value was in bold. We calculated hazaed ratios and *P* values with an adjusted multivariate Cox proportional hazards regression model, including age, sex, HBsAg, TNM stage, differentiation, serum AFP and mtDNA content as covariates.

### Prognostic prediction of leukocyte mtDNA content complementing to TNM stage

**Figure 2:**
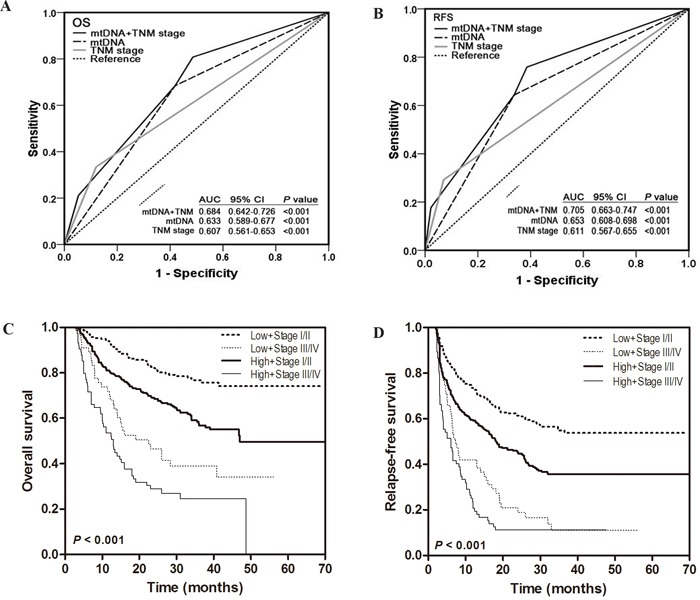
Joint prognostic values of leukocyte mtDNA content and TNM stage in HCC patients **A-B.** ROC curve analysis was used to compare the sensitivity and specificity for prediction of OS and RFS by the combined mtDNA content and TNM stage model, mtDNA content alone model and TNM stage alone model. **C-D.** Kaplan-Meier curves of OS and RFS in subgroups stratified by mtDNA content and TNM stage. Hazards ratios and 95% CIs were calculated by multivariate Cox proportional hazards regression model, adjusted for age, sex, HBsAg, differentiation, TNM stage, serum AFP and mtDNA content as covariates.

Considering prognosis heterogeneity in same TNM stage, we thus evaluated whether leukocyte mtDNA content can improve prognostic prediction based on TNM stage in the total HCC patient populations. As shown in Figure 2A and 2B, ROC analysis showed that the combined model of TNM stage and mtDNA had the largest AUC, indicating a significantly better prediction efficacy of OS and RFS than either TNM stage or mtDNA only model. We then compared the OS and RFS of HCC patients in different subgroups divided by TNM stage together with mtDNA content by Kaplan-Meier survival analysis and found that patients with high mtDNA content at TNM stage III/IV exhibited the worst OS and RFS, whereas those with low mtDNA content at TNM stage I/II had the best OS and RFS (both log-rank *P* < 0.001) (Figure 2C and 2D). In the multivariate Cox regression analysis, we found that patients with high mtDNA content at TNM stage III/IV had the highest risk of death and recurrence when using patients with low mtDNA content and at lower TNM stage I/II as reference (HR = 6.12, 95%CI = 4.17-8.98 for OS; HR = 4.29, 95%CI = 3.12 – 5.88 for RFS) (Supplementary Table 4).

### Immunophenotypes of PBMCs and plasma concentration of cytokines in HCC patients with different leukocyte mtDNA content

**Figure 3:**
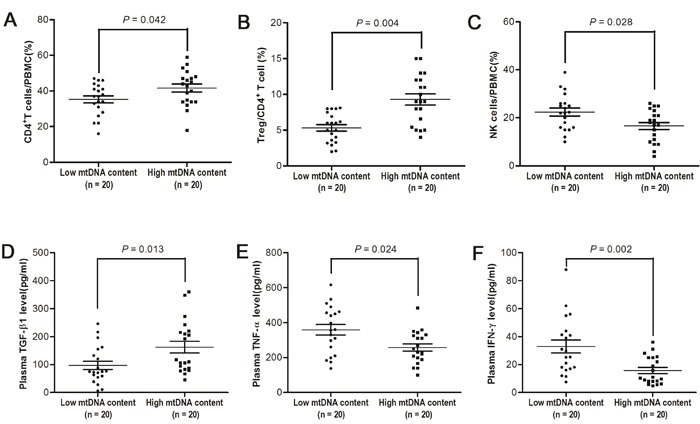
Immunophenotypes of PBMCs and plasma concentration of cytokines in HCC patients with different leukocyte mtDNA content **A-C.** Flow cytometry analyses for percentage of CD4^+^ in PBMCs, Treg cells in CD4^+^ T cells and NK-cells in PBMCs from HCC patients with high and low mtDNA content (both n = 20). **D-F.** ELISA analyses for the plasma concentrations of TGF-β1, TNF-α and IFN-γ from HCC patients with high and low mtDNA content (both n = 20).

To explore the potential mechanisms underlying leukocyte mtDNA content as a independent prognostic predictor for HCC patients, we first examined the associations between subtypes of lymphocytes in PBMCs and leukocyte mtDNA content in 40 HCC patients, whose demographic and clinical characteristics were summarized in supplementary Table 5. FACS analyses showed that patients with high leukocyte mtDNA content which was defined by using 0.98 as cutoff point had significantly higher percentage of CD4^+^ T cells (41.6% *vs.* 35.3%, *P* = 0.042, Figure 3A) and CD4^+^CD25^+^FOXP3^+^ regulatory T (Treg) cells (5.3% *vs.* 9.3%, *P* = 0.004, Figure 3B) and lower percentage of natural killer (NK) cells (22.4% *vs.* 16.6%, *P* = 0.028, Figure 3C) than those with low leukocyte mtDNA content. In addition, patients with high mtDNA content had significantly higher plasma TGF-β1 concentration (97.3 pg/ml *vs.* 162.1 pg/ml, *P* = 0.013, Figure 3D) and lower TNF-α and IFN-γ concentration (358.7 pg/ml *vs.* 258.0 pg/ml, *P* = 0.024 and 32.9 pg/ml *vs.* 15.7 pg/ml, *P* = 0.002, respectively, Figure 3E and 3F) than those with low mtDNA content. However, there was no significant difference in the frequency of other immune cells or the concentration of other cytokines between patients with high and low mtDNA content (Supplementary Figure 2).

### Elevated leukocyte mtDNA content induced an immunosuppressive phenotype by ROS-mediated secretion of TGF-β1

**Figure 4:**
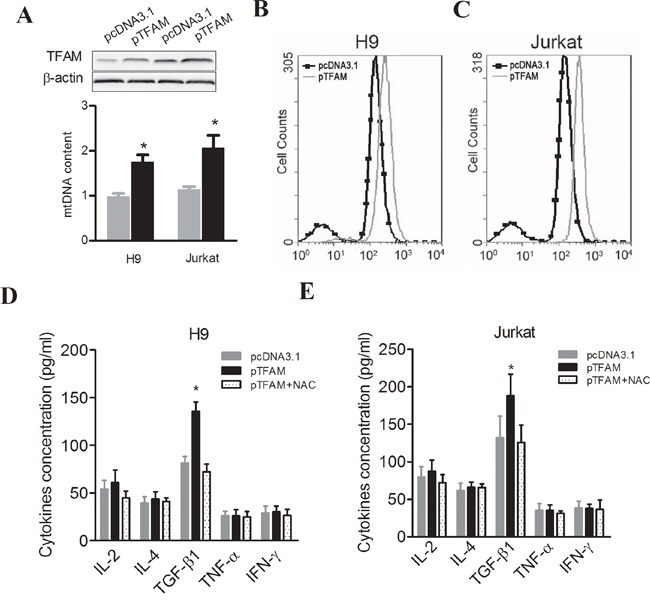
Elevated mtDNA content induced the production of ROS to increase the secretion of TGF-β1 in T cells **A.** TFAM expression at protein level detected by Western blot, and relative mtDNA content measured by real-time PCR-based method in H9 and Jurkat cells 48 hours after transfection with pTFAM or pcDNA3.1. **B.** and **C.** ROS measured by staining with H_2_DCF-DA and then analyzed by flow cytometer in H9 and Jurkat cells with different plasmid transfection or treatment. **D.** Cytokines in culture supernatants measured by ELISA in H9 cells with different plasmid transfection or treatment. **E.** Cytokines in culture supernatants measured by ELISA in Jurkat cells with different plasmid transfection or treatment. ^*^ *P*<0.05, compared with related controls.

**Figure 5:**
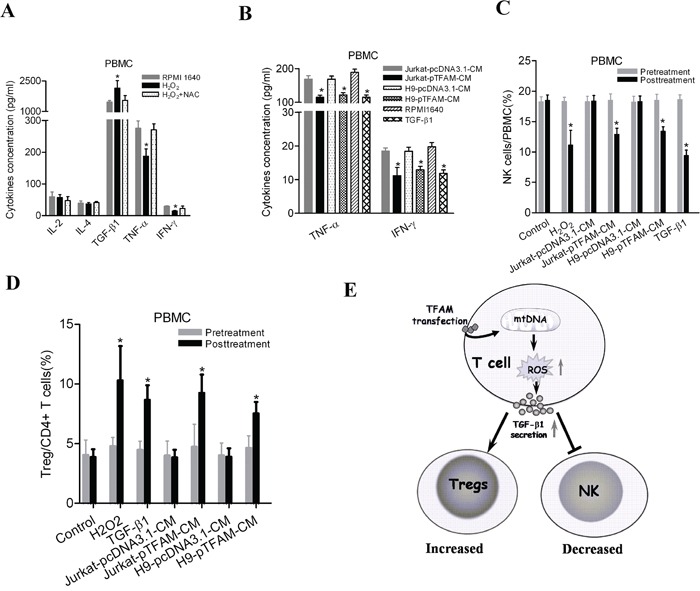
Elevated mtDNA content induced an immunosuppressive phenotype by ROS-mediated secretion of cytokines in PBMCs **A.** Concentration of cytokines in culture supernatants measured by ELISA in PBMCs with treatment of H_2_O_2_ (100μM) or both H_2_O_2_ (100μM) and NAC (20mM) for 12 hours. **B.** Concentration of TNF-α and IFN-γ measured by ELISA in PBMCs cultured with different conditional medium (CM). **C.** Frequency of NK cells from healthy donors measured by flow cytometry 48h after different treatments. 50ng/mL of TGF-β1 was used when appropriate. **D.** Frequency of Treg cells in CD4^+^ T cells from healthy donors measured by flow cytometry in different treatments. **E.** A schematic diagram on the underlying mechanism of mtDNA content-mediated immunosuppression. ^*^ *P*<0.05, compared with related controls.

To further identify the cause-effect relationship between mtDNA content variation and immunophenotypes of PBMCs or plasma concentration of cytokines, mitochondrial transcription factor TFAM was overexpressed to induce the elevation of mtDNA content in both Jurkat and H9 cells (Figure 4A). Then, intracellular ROS was found to be significantly increased in both cells (Figure 4B and 4C). We then measured the secretion of cytokines and found that TFAM overexpression (i.e. higher mtDNA content) significantly increased the concentration of TGF-β1 in culture supernatants of H9 and Jurkat, whereas the reducing reagent *N*-acetyl cysteine (NAC), a ROS scavenger, reversed this effect (Figure 4D and 4E). Moreover, H_2_O_2_ directly induced TGF-β1 secretion and suppressed TNF-α and IFN-γ secretion in PBMCs from healthy donors, while NAC significantly counteracted the effects of H_2_O_2_ (Figure 5A). TGF-β1 has been reported to suppresses the secretion of TNF-α, an important inhibitor of Treg activation [26], as well as the secretion of IFN-γ. Thus, we assessed the impact of culture supernatants from Jurkat or H9 cells with different mtDNA content on the PBMCs from healthy donors and found that supernatants from both TFAM-overexpressed Jurkat and H9 cells significantly inhibited TNF-α and IFN-γ secretion (Figure 5B). NK cell frequency was significantly decreased in PBMCs by H_2_O_2_ treatment or TGF-β1 treatment and culturing with supernatants from TFAM-overexpressed cells (Figure 5C). Furthermore, Treg frequency was significantly elevated in PBMCs by treatment with H_2_O_2_ or TGF-β1 and culturing with supernatants from TFAM-overexpressed cells (Figure 5D). All our findings provide the evidence that higher mtDNA content induces the quantitative variation of Treg and NK cells via ROS-mediated secretion of TGF-β1, thereby contributing to the worse prognosis of HCC (Figure 5E).

## DISCUSSION

A growing number of epidemiological studies have demonstrated that leukocyte mtDNA content is closely related to risk of various cancers. For instance, several previous studies have reported that leukocyte mtDNA content is associated with the risks of breast, pancreas, liver, esophageal, colon and lung cancers [19, 20, 27, 28]. Moreover, Xia et al. have demonstrated that leukocyte mtDNA content is associated with the T stage of breast cancer, indicating that high mtDNA content may facilitate the progression of cancer [21]. In this study, we for the first time identified leukocyte mtDNA content as an independent prognostic factor for HCC, indicating that high leukocyte mtDNA content was associated with poor OS and RFS of patients. In comparison, Hashad et al. have reported that HCV-related HCC patients with multicentric hepatic lesions have significantly lower mtDNA content than those with less advanced disease, suggesting that functional roles of mtDNA copy number may be disease-specific [16]. Collectively, these findings support the point of view that mtDNA content variations are involved in the progression of cancer, although the molecular mechanisms underlying the regulation of mtDNA copy number, especially in lymphocytes from cancer patients, remains to be largely unclear.

Current TNM staging system plays an important role in the prognosis prediction and treatment decision-making of HCC patients. However, patients in the same stage undergoing same therapeutic regimen show heterogeneous outcome. Therefore, introduction of novel biomarkers into the TNM staging system may improve the prognosis prediction of HCC patients. As shown in our study, integration of leukocyte mtDNA into TNM stage-based prognosis prediction models significantly improved the prediction power of overall survival of HCC patients. Once HCC patients with poor prognosis can be effectively predicted, potential therapeutic interventions will be applied, such as frequent imaging scan (CT or MRI) and AFP level monitoring, treatment with adjuvant transarterial chemoembolization (TACE) or Sorafenib (an orally active multikinase inhibitor) [29].

The biological mechanism behind the association between leukocyte mtDNA content alteration and HCC progression remains to be clarified. Previous studies have demonstrated that either increase in Tregs or decrease in NK cell frequency is significantly associated with poor survival of HCC patients [30]. It is generally considered that NK cells exert immunosurveillance against cancer cells, while Tregs inhibit anticancer immunity. In present study, we found that HCC patients with high leukocyte mtDNA content had an increased percentage of Tregs and the decreased percentage of NK-cells in peripheral blood, suggesting that mtDNA content may be involved in the immunosuppression of HCC patients. Moreover, it has been reported that IFN-γ and TNF-α levels in plasma are associated good prognosis of HCC [31, 32], while TGF-β1 plasma concentration is associated with poor prognosis of HCC [33]. IFN-γ is one of the key mediator of cytotoxicity secreted by NK cells, while TNF-α enhances the functions of NK cells and suppress the functions of Tregs. As an important immunosuppressive cytokine, TGF-β1 has a wide spectrum of inhibitory functions, including promoting Treg proliferation and differentiation, as well as inhibiting the expansion and functions of helper T cells and killer cells. In this study, our data also showed that HCC patients with high leukocyte mtDNA content had lower plasma TNF-α and IFN-γ but higher TGF-β1 concentrations, indicating that mtDNA content may affect cytokine secretion of immune cells.

In mammalian cells, mitochondria are the major source of reactive oxygen species (ROS) [34]. Generally, the increase in mtDNA content is related to an increase of ROS production [35]. Previous studies have reported that mitochondrial ROS is an important inducer of TGF-β1 [36], a key cytokine accounting for the differentiation, proliferation and functions of Treg and NK cells. Consistently, our in vitro cell model analysis indicated that high mtDNA content induced ROS generation to increase the secretion of TGF-β1 in T cell lines. Mitochondria-derived ROS play an important role in the immune functions of T cells, such as activation of naive T cells, cytokine secretion of effect T cells, and apoptosis of activated T cells [37]. Kraaij et al. have showed that abundant ROS production could lead to increased peripheral Tregs, an important suppressor of anti-tumor immunity [38]. In line with these evidence, we also found that supernatants from Jurkat and H9 cells with higher mtDNA content increased the frequency of Tregs in PBMCs. Previous data have shown that high concentration of TGF-β1 and low concentration of TNF-α facilitate the differentiation and expansion of Tregs [39, 40]. Therefore, it is possible that elevated mtDNA content induces ROS production and thus affect the secretion of cytokines, leading to increased Tregs in PBMCs. Furthermore, ROS can also induce NK cell dysfunction and apoptosis [41], and oxidative stress has been shown to induce the decrease of NK cell in PBMCs. In line with these findings, we confirmed that H_2_O_2_ or supernatants from Jurkat and H9 cells induced the decrease of NK cell frequency in PBMCs, probably via increased Tregs and TGF-β1. Collectively, these data suggest that elevated mtDNA content in leukocytes might promote the progression of HCC via ROS-induced immunosuppressive effects, which may explain the poor prognosis of these patients.

In summary, our findings indicate that leukocyte mtDNA content is an independent prognosis marker for HCC and can improve the prediction of TNM stage-based prognosis models. Mechanically, high mtDNA content may contribute to ROS-mediated immunosuppressive phenotype in the peripheral blood lymphocytes. Our study provides new insight into HCC pathological progression.

## MATERIALS AND METHODS

### Patient population, sample and clinical data collection

The eligibility criteria for patient recruitment were set as follows: (1) histologically-confirmed hepatocellular carcinoma (HCC); (2) receiving curative surgery; (3) only Child-Pugh A classification; (4) availability of complete clinical and follow-up data; (5) no preoperative anticancer treatment; (6) no history of other malignancy; and (7) alive at least 1 months after surgery. Finally, a total of 618 surgical HCC patients from two independent cohorts were included in the present study. Training cohort (n = 113) was collected between March 2008 and September 2012 at Xijing Hospital affiliated with Fourth Military Medical University (FMMU) in Xi'an, China. Validation cohort (n = 505) was collected between September 2009 and July 2013 at Eastern Hepatobiliary Surgery Hospital affiliated with Secondary Military Medical University (SMMU) in Shanghai, China. Before surgery, 5 ml venous blood was collected from each patient and used for DNA extraction using the E.Z.N.A. blood DNA Midi Kit (Omega Bio-Tek, Norcross, GA). In addition, 40 additional HCC patients were enrolled between May 2014 and October 2014 from Tangdu Hospital of FMMU for immunoassays and 5 ml venous blood was collected from each patient and separated by centrifugation. The plasma was used for cytokine assay and the blood cells were used for DNA extraction or isolation of peripheral blood mononuclear cells (PBMCs) by density gradient centrifugation over Ficoll-Hypaque (Amersham Pharmacia Biotech, NJ) as previously described [23]. PBMCs were also collected from 3 healthy donors from our lab. The demographic information, clinical and follow-up data of each patient was collected by well-trained staff interviewers or clinical specialists and described in Supplementary Methods. The latest follow-up date was January 2014 and the median follow-up duration was 33.4 months (ranging from 3.6 to 70 months). Overall survival was defined as the interval from surgery to death or last follow-up. Recurrence-free survival (RFS) was defined as the time from surgery to the date of the first recurrence or distant metastasis of HCC or death, whichever occurred first. The study was approved by the Ethic Committee of the Fourth Military Medical University and written informed consent was obtained from all participants.

### Detection of mtDNA content by real-time quantitative PCR

Relative mtDNA content was measured by a two-step real-time quantitative PCR-based method. In the first step, the ratio of mtDNA copy number to HGB copy number was determined for each sample from standard curves. In the second step, the mtDNA/HGB ratio for each sample was normalized to a calibrator DNA to standardize between different runs and the normalized mtDNA/HGB ratio was defined as the measurement of relative mtDNA content, which was affected by DNA sample used for standard curve, therefore only can be compared in this study. The detailed information was provided in Supplementary Methods.

### Cell culture

T lymphoblast cell lines Jurkat and H9 were routinely maintained in RPMI 1640 medium supplemented with 10% FBS (Thermo Scientific Hyclone). Cells were first activated by 1μg/ml phytohemagglutinin (PHA) for 12 h, washed, and subsequently cultured in RPMI 1640 medium for 24 h. Then, the culture supernatant was filtered and collected for further immunoassays or used as conditioned medium (CM) for PBMC culturing. Fresh PBMCs were isolated from healthy donors. Conditioned culturing was performed in RPMI 1640 medium with 10% heat-inactivated FBS and 30% culture supernatant from Jurkat or H9 cells. After 24 h, cultured PBMCs and supernatant were collected and used for immunoassays.

### Plasmid construction and cell transfection

The full-length DNA sequence encoding mitochondrial transcription factor A (TFAM) was cloned into pcDNA3.1(+) vector (defined as pTFAM) as previously described [24]. The recombinant or control plasmid was transfected into Jurkat or H9 cells using Lipofectamine 2000 reagent (Life Sciences) according to the manufacturer's instructions. The overexpression of TFAM was confirmed by Western blotting as described in Supplementary Methods.

### Immunophenotype analysis of PBMCs by flow cytometry

Fresh or cultured PBMCs were fixed with 4% formaldehyde and stained with fluorescence-conjugated antibodies against the following immune markers: CD3, CD4, CD8, CD25, FOXP3, CD19 and CD56 (BD Biosciences, NJ). Appropriate isotype controls were included for each sample. The immunophenotype detection was performed on a FACScan flow cytometer (Becton Dickinson, Franklin Lakes, NJ).

### Concentration detection of cytokines by enzyme-linked immunoassay (ELISA)

The concentrations of interleukin (IL)-2, IL-4, transforming growth factor (TGF)-β1, tumor necrosis factor (TNF)-α and interferon (IFN)-γ were detected in plasma of HCC patients or culturing supernatant by using ELISA kits (eBioscience, San Diego, CA) according to the manufacturer's instructions.

### Detection of ROS by flow cytometry

Cellular reactive oxygen species (ROS) were detected by the fluorescent probe H_2_DCFH-DA (Beyotime, shanghai, China) according to the manufacturer's protocols. Briefly, H_2_DCFH-DA was diluted to a final concentration of 10 μM with serum free medium. Then suspension of H9 and Jurkat cells transfected with different expression plasmids was incubated with DCFH- DA at 37°C for 30 minutes and then analyzed on a FACScan flow cytometer (Becton Dickinson, Franklin Lakes, NJ) with an excitation wavelength of 488 nm and an emission wavelength of 535 nm.

### Statistical analysis

Chi-squared test was used to examine differences of categorical variables between subgroups. Student's *t*-test was used to analyze the difference of normally distributed continuous variables between two groups, while Mann-Whitney U test and Kruskal-Wallis H test were employed for the comparison of abnormally distributed continuous variables. The receiver operating characteristic curve analysis was used to select a leukocyte mtDNA content cutoff point for OS and RFS in the training cohort as previously described [25]. Kaplan-Meier survival curve was plotted and compared with a log-rank test. Multivariate Cox proportional hazards regression model was used to calculate the hazard ratio and 95% confidence interval for the association of clinicopathological variables and mtDNA content with OS and RFS of HCC patients. All statistical analyses were performed using the IBM SPSS Statistics 19.0 software (IBM), and *P* < 0.05 was considered statistically significant.

## SUPPLEMENTARY METHODS FIGURES AND TABLES


