# Supplementary material for: High leukocyte mtDNA content contributes to poor prognosis through ROS-mediated immunosuppression in hepatocellular carcinoma patients
Source: Oncotarget. 2016 Mar 14;7(16):22834–45. doi: 10.18632/oncotarget.8071 (PMC5008404; doi:10.18632/oncotarget.8071)
Supplement: Supplementary file 1 [file oncotarget-07-22834-s001.pdf]

SUPPLEMENTARY METHODS

Detection of mtDNA content by real-time quantitative PCR

Relative leukocyte mtDNA content was measured by a two-step real-time quantitative PCR-based method as previously described [1]. Briefly, two pairs of primers were used in the two steps of relative quantification for mtDNA content. The primer sequences for the mitochondrial ND1 gene were as follows: forward primer (ND1-F), 5'-CCCTAAAACCCgCCACATCT-3'; reverse primer (ND1-R), 5'-GAGCGATGGTGAGAGCTAAGGT-3'. The single-copy nuclear gene human globulin (HGB) was used as a housekeeping gene control and the primer pair was as follows: forward primer (HGB-1), 5'-GTGCACCTGACTCCTGAGGAGA-3'; reverse primer (HGB-2), 5'-CCTTGATACCAACCTGCCCAG-3'. In the first step, the ratio of mtDNA copy number to HGB copy number was determined for each sample from standard curves. In the second step, the mtDNA/HGB ratio for each sample was normalized to a calibrator DNA to standardize between different runs and the normalized mtDNA/HGB ratio was defined as the measurement of relative mtDNA copy number in this study. The calibrator DNA is a genomic DNA sample from a healthy control subject. It was measured in each run and used to standardiszedifferent real-time quantitative PCR runs. The value of this only scaling factor varied from 0.89 to 1.17 across different runs. The intra-assay or interassay variations were evaluated by assaying one sample in eight replicates or in three different runs, respectively.

The PCR reaction (20 µL) consisted of 1 × SYBR green mastermix (TaKaRa, Dalian, China), 10 nM ND1-R (or HGB-1) primer, 10 nM ND1-F (or HGB-2) primer, and 8 ng of genomic DNA. The thermal cycling conditions for both primer pairs were 95°C for 30 sec, followed by 35 cycles of 94°C for 30 sec, 58°C for 30 sec, and 72°C for 50 sec with signal acquisition. The PCRs were always performed on separate 96-well plates, with the same samples in the same well positions. All samples were assayed in duplicate using the Mx3005P QPCR System (Agilent, Santa Clara, CA). In each run, negative and positive controls, a calibrator DNA, and a standard curve were included. The R^2 for each standard curve was ≥ 0.99, with acceptable standard deviations set under 0.25 (for the Ct values). Otherwise, the test was repeated. The reproducibility of the assay was assessed by analyzing the intra- and inter-assay coefficient of variation. The mean inter-assay coefficient of variation (CV) was 8.1% (range, 5.2 % to 11.7 %), whereas the mean intra-assay CV was 6.2% (range, 4.4 % to 9.1 %), indicating excellent assay reproducibility. All persons conducting this experimental procedures were blinded to the endpoint of our study.

Western blotting

Protein extracts (20µg) from Jurkat cells and H9 cells at 24h after transfection with empty vector pcDNA3.1 or Tfam expression vector pTFAM were prepared using lysis buffer (10 mM HEPES, pH 7.9, 10 mM KCl, 2 mM $MgCl_2$, 0.5 mM dithiothreitol, 1 mM PMSF, 5 µg/mL aprotinin, 5 µg/mL pepstatin, 5 µg/mL leupeptin, and 1% Triton X-100), then separated by 10% SDS-PAGE, and analyzed by Western blot using rabbit anti-human TFAM polyclonal antibodies (Abcam, ab47517, 1:1000) and an enhanced chemiluminescence system (ECL, Amersham Pharmacia Biotech). Equivalent protein loading was verified by blotting with anti-β-actin antibody (Abcam, ab119716, 1:5000).

REFERENCES

1. Xing J, Chen M, Wood CG, Lin J, Spitz MR, Ma J, Amos CI, Shields PG, Benowitz NL, Gu J, de Andrade M, Swan GE and Wu X. Mitochondrial DNA content: its genetic heritability and association with renal cell carcinoma. Journal of the National Cancer Institute. 2008; 100:1104-1112.

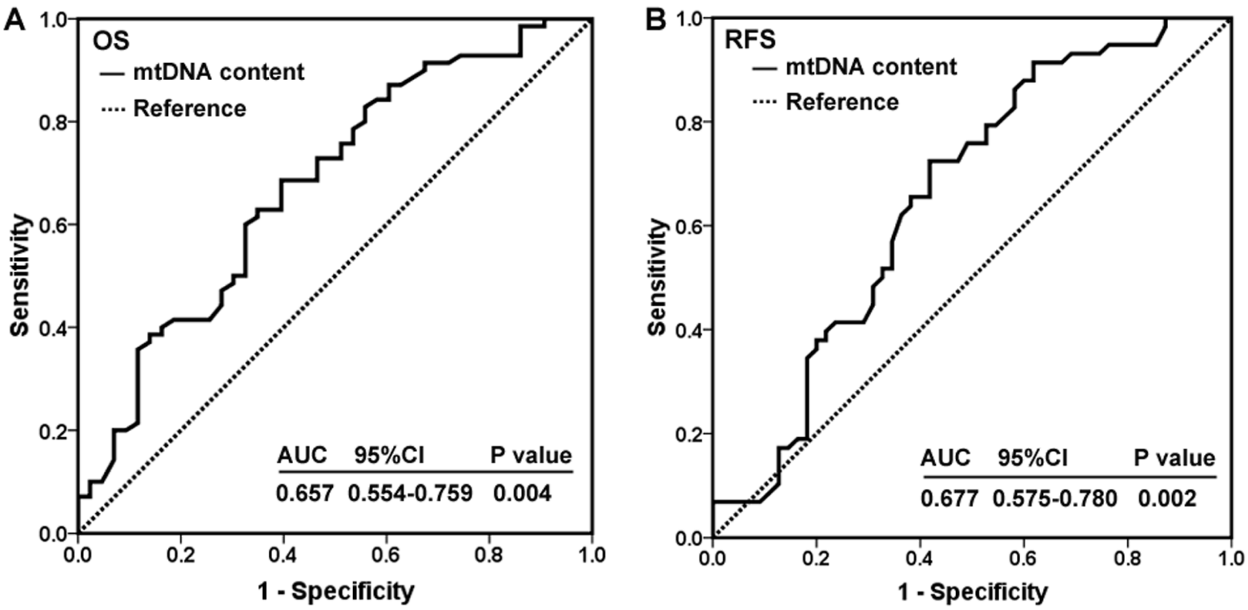

Supplementary Figure 1: Receiver operating characteristic (ROC) curve analysis of mtDNA content cutoff point in training cohort for. A. OS and **B.** RFS.

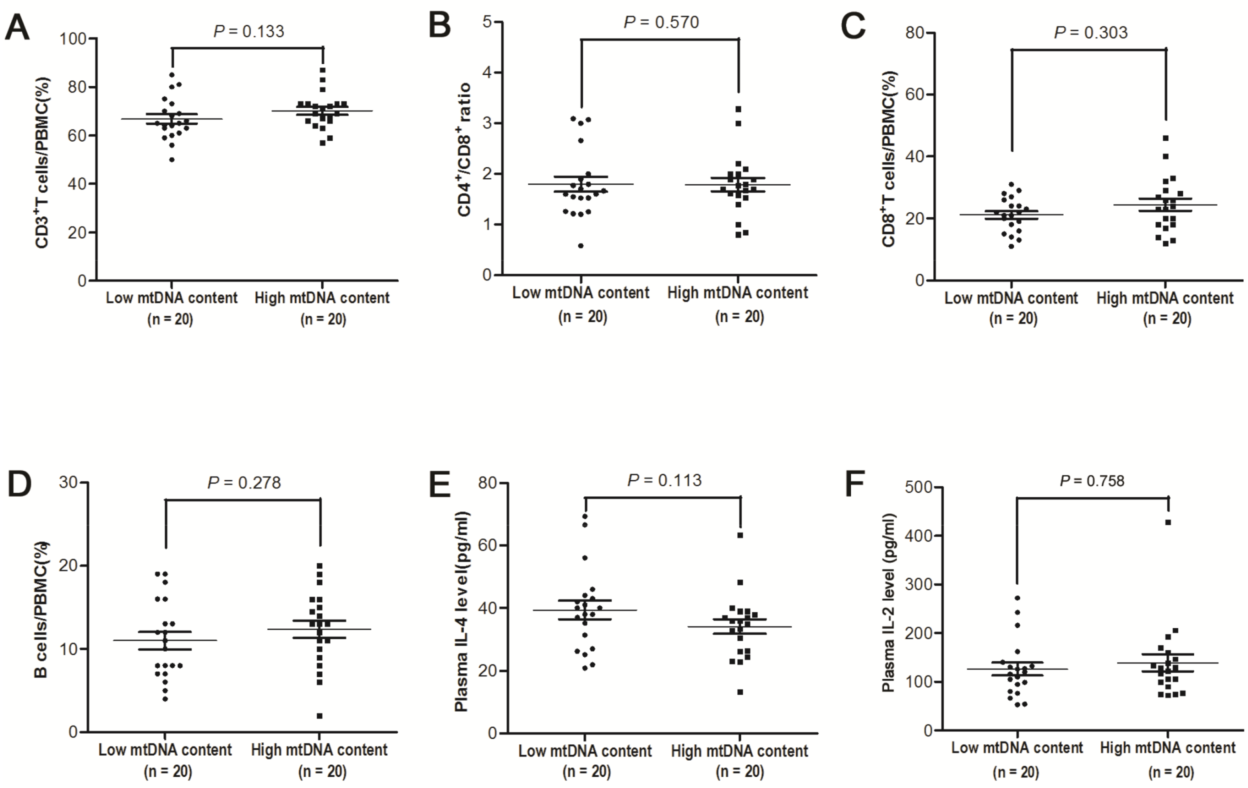

Supplementary Figure 2: Immunophenotypes of PBMCs and plasma concentration of cytokines in HCC patients with different leukocyte mtDNA content. A-D. Flow cytometry analyses for percentage of $CD3^+$ in PBMCs, $CD4^+$ /$CD8^+$ T cells ratio, $CD8^+$ T cells in PBMCs and B cells in PBMCs from HCC patients with high and low mtDNA content (both n = 20). **E-F.** ELISA analyses for the plasma concentrations of IL-4 and IL-2 from HCC patients with high and low mtDNA content (both n = 20).

Supplementary Table 1: Demographic and clinical characteristics of HCC patient population

Variables	Total cohort (n = 618)	Training cohort (n = 113)	Validation cohort (n = 505)	*P* value
	Number (%)	Number (%)	Number (%)	
**Sex**				
Male	544 (88.0)	103 (91.2)	441 (87.3)	0.258
Female	74 (12.0)	10 (8.8)	64 (12.7)	
**HBsAg**				
Positive	559 (90.5)	99 (87.6)	460 (91.1)	0.255
Negative	59 (9.5)	14 (12.4)	45 (8.9)	
**Tumor size(cm)**				
<6	322 (52.1)	71 (62.8)	251 (49.7)	0.012
≥6	296 (47.9)	42 (37.2)	254 (50.3)	
**Number of tumors**				
Single	503 (81.4)	92 (81.4)	411 (81.4)	0.994
Multiple	115 (18.6)	21 (18.6)	94 (18.6)	
**PVT**				
Yes	66 (10.7)	13 (11.5)	53 (10.5)	0.753
No	552 (89.3)	100 (88.5)	452 (89.5)	
**TNM stage**				
I	424 (68.6)	62 (54.9)	362 (71.7)	<0.001
II	64 (10.4)	20 (17.7)	44 (8.7)	
III	115 (18.6)	29 (25.7)	86 (17.0)	
IV	15 (2.4)	2 (1.7)	13 (2.6)	
**Differentiation, n(%)**				
Well	28 (4.5)	12 (10.6)	16 (3.2)	<0.001
Moderate	185 (29.9)	43 (38.1)	142 (28.1)	
Poor	405 (65.6)	58 (51.3)	347 (68.7)	
**Serum AFP(μg/L)**				
<200	237 (38.3)	33 (29.2)	204 (40.4)	0.027
≥200	381 (61.7)	80 (70.8)	301 (59.6)	
**Recurrence**				
Yes	373 (60.4)	73 (64.6)	300 (59.4)	0.017
No	245 (39.6)	40 (35.4)	205 (40.6)	
**Death**				
Yes	240 (38.8)	58 (51.3)	182 (36.0)	0.001
No	378 (61.2)	55 (48.7)	323 (64.0)	
**Median age by years (range)**	52 (21 - 79)	51 (27 -77)	52 (21 - 79)	0.093
**mtDNA content, Median(range)**	0.98 (0.12 - 3.70)	0.99 (0.16 - 3.39)	0.98 (0.12 - 3.70)	0.987
**Follow-up by months, Median(range)**	33.4 (3.6 - 70.0)	44.6 (16.0 - 70.0)	32.2 (3.6 - 56.0)	<0.001

Supplementary Table 2: Comparison of mtDNA content between different subgroups of HCC patients in the training, validation and combined cohorts

Characteristics	Training cohort (n = 113)		Validation cohort (n = 505)		Combined cohort (n = 618)	
	mtDNA content (median)	*P* value	mtDNA content (median)	*P* value	mtDNA content (median)	*P* value
**Age, years**						
≤ 52	1.00 (0.31 - 3.27)		0.99 (0.21 - 3.70)		0.99 (0.21 - 3.70)	
> 52	0.95 (0.29 - 2.89)	0.186	0.98 (0.23 - 3.57)	0.697	0.97 (0.23 - 3.57)	0.335
**Sex**						
Male	0.99 (0.29 - 3.27)		0.99 (0.21 - 3.70)		0.99 (0.21 - 3.70)	
Female	0.89 (0.31 - 2.20)	0.547	0.95 (0.22 - 2.79)	0.418	0.94 (0.22 - 2.79)	0.870
**HBsAg**						
Positive	0.99 (0.29 - 3.27)		0.99 (0.21 - 3.70)		0.99 (0.21 - 3.70)	
Negative	0.89 (0.63 - 1.21)	0.164	0.94 (0.45 - 2.79)	0.863	0.93 (0.45 - 2.79)	0.526
**Tumor size(cm)**						
<6	0.99 (0.38 - 2.60)		0.94 (0.21 - 3.70)		0.96 (0.21 - 3.70)	
≥6	0.92 (0.29 - 3.27)	0.452	1.00 (0.28 - 3.67)	0.121	0.99 (0.28 - 3.67)	0.266
**Number of tumors**						
Single	0.96 (0.29 - 2.63)		0.97 (0.21 - 3.70)		0.97 (0.21 - 3.70)	
Multiple	1.00 (0.59 - 3.27)	0.107	1.00 (0.31 - 3.07)	0.624	1.00 (0.31 - 3.27)	0.412
PVT						
Yes	1.01 (0.76 - 3.27)		1.05 (0.21 - 2.55)		1.03 (0.21 - 3.27)	
No	0.95 (0.29 - 2.89)	0.34	0.96 (0.22 - 3.70)	0.908	0.96 (0.22 - 3.70)	0.419
**TNM stage**						
I+II	0.96 (0.55 - 2.89)		0.98 (0.22 - 3.70)		0.98 (0.22 - 3.70)	
III+IV	1.05 (0.29 - 3.27)	0.092	1.00 (0.21 - 3.07)	0.625	1.01 (0.21 - 3.27)	0.322
**Differentiation**						
Well+Moderate	0.93 (0.29 - 3.27)		0.97 (0.23 - 3.67)		0.96 (0.23 - 3.67)	
Poor	1.00 (0.38 - 2.60)	0.996	0.99 (0.21 - 3.70)	0.712	0.99 (0.21 - 3.70)	0.559
**Serum AFP(μg/L)**						
<200	0.99 (0.29 - 3.27)		0.99 (0.21 - 3.70)		0.99 (0.21 - 3.70)	
≥200	0.94 (0.31 - 2.56)	0.412	0.96 (0.22 - 2.79)	0.679	0.96 (0.22 - 2.79)	0.661
**Recurrence**						
Yes	1.05 (0.55 - 3.27)		1.04 (0.21 - 3.57)		1.04 (0.21 - 3.57)	
No	0.87 (0.29 - 2.20)	**<0.001**	0.88 (0.22 - 3.70)	**0.003**	0.87 (0.22 - 3.70)	**0.002**
**Death**						
Yes	1.05 (0.59 - 3.27)		1.07 (0.21 - 3.57)		1.07 (0.21 - 3.57)	
No	0.80 (0.29 - 2.56)	**<0.001**	0.90 (0.22 - 3.70)	**0.001**	0.88 (0.22 - 3.70)	0.001

Notes: Data were presented as Median (Range), unless otherwise stated. TNM, tumor-node-metastasis; mtDNA content, mitochondrial DNA content. The P values were calculated using an unpaired student's t test.

Supplementary Table 3: Univariate Cox regression analysis of overall survival and recurrence-free survival for HCC patients

Variables	Training cohort		Validation cohort		Pooled analysis	
	HR (95% CI)	*P* value	HR (95% CI)	*P* value	HR (95% CI)	*P* value
**Overall survival**						
Age (>52 vs. ≤52)	1.12 (0.81 - 1.69)	0.662	1.19 (0.87 - 1.64)	0.283	1.11 (0.85 - 1.44)	0.441
Sex (male *vs.* female)	2.06 (0.75 - 5.65)	0.159	1.35 (0.81 - 2.23)	0.247	1.48 (0.94 - 2.31)	0.089
HBsAg (postive *vs.* negative)	1.49 (0.95 - 3.48)	0.086	1.32 (0.70 - 2.51)	0.657	1.14 (0.74 - 1.75)	0.568
Tumor size (≥6cm *vs.* <6cm)	2.90 (1.83 - 4.60)	<0.001	2.29 (1.63 - 3.22)	<0.001	2.40 (1.84 - 3.15)	<0.001
Number of tumors (multiple *vs.* single)	1.90 (1.16 - 3.10)	0.011	2.11 (1.47 - 3.04)	<0.001	2.04 (1.52 - 2.73)	<0.001
PVT (yes *vs.* no)	5.87 (3.24 - 10.64)	<0.001	4.41 (2.99 - 6.49)	<0.001	4.74 (3.43 - 6.55)	<0.001
TNM stage (III+IV *vs.* I+II)	4.18 (2.62 - 6.66)	<0.001	3.32 (2.36 - 4.66)	<0.001	3.55 (2.70 - 4.67)	<0.001
Differentiation (Poor *vs.* Moderate+Well)	2.96 (1.80 - 4.84)	<0.001	2.30 (1.52 - 3.48)	<0.001	2.46 (1.79 - 3.37)	<0.001
Serum AFP (≥200μg/L *vs.* <200μg/L)	2.05 (1.27 - 3.29)	0.003	2.26 (1.63 - 3.12)	<0.001	2.14 (1.65 - 2.79)	<0.001
mtDNA content (high *vs.* low)	1.99 (1.27 - 3.54)	0.001	1.41 (1.02 - 1.93)	0.042	1.53 (1.17 - 2.00)	0.002
**Recurrence-free survival**						
Age (>52 *vs.* ≤52)	1.06 (0.62 - 1.71)	0.722	1.09 (0.72 - 1.37)	0.836	1.08 (0.71 - 1.34)	0.808
Sex (male *vs.* female)	1.09 (0.41 - 2.03)	0.911	1.17 (0.79 - 1.64)	0.523	1.13 (0.73 - 1.88)	0.381
HBsAg (postive *vs.* negative)	1.43 (0.77 - 2.36)	0.202	1.28 (0.84 - 1.57)	0.917	1.38 (0.79 - 1.96)	0.544
Tumor size (≥6cm *vs.* <6cm)	1.94 (1.19 - 3.28)	0.008	1.85 (1.43 - 2.89)	<0.001	1.91 (1.55 - 2.94)	<0.001
Number of tumors (multiple *vs.* single)	2.23 (1.16 - 4.09)	0.011	1.61 (1.12 - 2.34)	0.013	1.83 (1.20 - 2.58)	0.004
PVT (yes *vs.* no)	2.44 (1.18 - 4.06)	0.009	1.91 (1.13 - 2.88)	0.012	2.07 (1.19 - 3.24)	0.008
TNM stage (III+IV *vs.* I+II)	4.48 (2.51 - 8.82)	<0.001	2.66 (1.63 - 5.72)	<0.001	2.92 (1.83 - 5.33)	<0.001
Differentiation (Poor *vs.* Moderate+Well)	2.58 (1.51 - 4.43)	<0.001	2.07 (1.28 - 4.18)	<0.001	2.16 (1.35 - 3.96)	<0.001
Serum AFP (≥200μg/L *vs.* <200μg/L)	1.99 (1.16 - 3.55)	0.009	1.86 (1.33 - 3.11)	<0.001	1.91 (1.22 - 2.93)	<0.001
mtDNA content (high *vs.* low)	1.98 (1.15 - 3.54)	0.01	1.93 (1.38 - 2.82)	<0.001	1.94 (1.26 - 2.98)	<0.001

Notes: mtDNA, mitochondrial DNA; HR, hazard ratio; 95% CI, 95% confidence interval.

Supplementary Table 4: Joint effects of mtDNA content and TNM stage on HCC patients' overall and recurrence-free survival

Variables	Overall survival		Recurrence-free survival	
	HR (95%CI)	*P* value	HR (95%CI)	*P* value
Low mtDNA content + Stage I/II	1 (reference)		1 (reference)	
Low mtDNA content + Stage III/IV	3.74 (2.40 - 5.81)	4.72×10^{-9}	3.03 (2.14 - 4.29)	4.07×10^{-10}
High mtDNA content + Stage I/II	2.03 (1.46 - 2.84)	3.12×10^{-5}	1.71 (1.33 - 2.19)	2.59×10^{-5}
High mtDNA content + Stage III/IV	6.12 (4.17 - 8.98)	2.23×10^{-20}	4.29 (3.12 - 5.88)	2.09×10^{-19}
P for trend		2.43×10^{-20}		4.87×10^{-20}

Notes: mtDNA, mitochondrial DNA; HR: hazard ratio; 95% CI, 95% confidence interval. We calculated hazaed ratios and *P* values with an adjusted multivariate Cox proportional hazards regression model, including age, sex, HBsAg, TNM stage, differentiation, serum AFP and mtDNA content as covariates.

Supplementary Table 5: Comparison of mtDNA content between different subgroups of 40 additional HCC patients

Characteristics	mtDNA content, median (range)	*P* value
**Age (years)**		
≤52	0.99 (0.21-3.70)	0.454
>52	0.97 (0.23-3.57)	
**Sex**		
Male	0.99 (0.21-3.70)	0.542
Female	0.94 (0.22-2.79)	
**HBsAg**		
Positive	0.99 (0.21-3.70)	0.638
Negative	0.94 (0.45-2.79)	
Tumor size(cm)		
<6	0.96 (0.21-3.70)	0.035
≥6	1.01 (0.28-3.67)	
Number of tumors		
Single	0.97 (0.21-3.70)	0.163
Multiple	1.00 (0.31-3.27)	
**PVT**		
Yes	1.05 (0.21-3.27)	0.109
No	0.96 (0.22-3.70)	
**TNM stage**		
I+II	0.97 (0.22-3.70)	0.258
III+IV	1.02 (0.21-3.27)	
**Differentiation**		
Well+Moderate	1.01 (0.21-3.70)	0.109
Poor	0.98 (0.22-3.67)	
**Serum AFP(μg/L)**		
<200	0.99 (0.21-3.70)	0.095
≥200	0.94 (0.22-3.27)	

TNM, tumor-node-metastasis; mtDNA content, mitochondrial DNA content. The *P* values were calculated using Mann-Whitney U test.
